# Development of estrogen receptor beta binding prediction model using large sets of chemicals

**DOI:** 10.18632/oncotarget.21723

**Published:** 2017-10-10

**Authors:** Sugunadevi Sakkiah, Chandrabose Selvaraj, Ping Gong, Chaoyang Zhang, Weida Tong, Huixiao Hong

**Affiliations:** ^1^ Division of Bioinformatics and Biostatistics, National Center for Toxicological Research, U.S. Food and Drug Administration, Jefferson, AR, USA; ^2^ Environmental Laboratory, U.S. Army Engineer Research and Development Center, Vicksburg, MS, USA; ^3^ School of Computer Science, University of Southern Mississippi, Hattiesburg, MS, USA

**Keywords:** decision forest, estrogen receptor, QSAR, Mold^2^, predictive model

## Abstract

We developed an ER_β_ binding prediction model to facilitate identification of chemicals specifically bind ER_β_ or ER_α_ together with our previously developed ER_α_ binding model. Decision Forest was used to train ER_β_ binding prediction model based on a large set of compounds obtained from EADB. Model performance was estimated through 1000 iterations of 5-fold cross validations. Prediction confidence was analyzed using predictions from the cross validations. Informative chemical features for ER_β_ binding were identified through analysis of the frequency data of chemical descriptors used in the models in the 5-fold cross validations. 1000 permutations were conducted to assess the chance correlation. The average accuracy of 5-fold cross validations was 93.14% with a standard deviation of 0.64%. Prediction confidence analysis indicated that the higher the prediction confidence the more accurate the predictions. Permutation testing results revealed that the prediction model is unlikely generated by chance. Eighteen informative descriptors were identified to be important to ER_β_ binding prediction. Application of the prediction model to the data from ToxCast project yielded very high sensitivity of 90-92%. Our results demonstrated ER_β_ binding of chemicals could be accurately predicted using the developed model. Coupling with our previously developed ER_α_ prediction model, this model could be expected to facilitate drug development through identification of chemicals that specifically bind ER_β_ or ER_α_.

## INTRODUCTION

Estrogen receptor (ER) is the ligand-dependent transcriptional factor. ER belongs to the nuclear receptor family. ER_α_ and ER_β_ are the two major isoforms reported for ER [1–3]. The two isoforms have different functions with various concentrations in tissues. Compared to ER_α_, ER_β_ has wider tissue distribution [4]. ER_β_ has similar structure architecture with other nuclear receptor proteins and contains 3 distinct domains: (i) N-terminal domain (NTD), (ii) DNA-binding domain (DBD), and (iii) Ligand-binding domain (LDB) or C-terminal domain (CTD). The NTD and LBD contain ligand responsive transcriptional activation function 1 (AF1) and activation function 2 (AF2) domains. The AF1 and AF2 domains are responsible for the regulation of the transcriptional activity of ER_β_ [5]. ER_β_ DBD and LBD had shown more than 95% and 55% sequence similarity with ER_α_ DBD and LBD, respectively. The ER_β_ NTD is shorter than the ER_α_ NTD and shows a very low sequence homology [6]. ER_β_ presents in both the nucleus and cytoplasm of the normal and cancer cells while ER_α_ which presents in nuclei of benign and cancer cells. The cytoplasmic ER_β_ binds with the estradiol or agonist and moves into the nucleus to form homo-dimer and then binds with the specific estrogen response elements (EREs) to activate the transcription process through the interaction between the transcriptional modulators and recruitment of the general transcriptional machinery [7]. ER_β_ may act as a marker in various types of cancers and a significant predictor in the breast cancer treated with tamoxifen [8]. ER_β_ is a potential cancer target, highly expressed in various cancers which are reported as a negative for ER_α_ [9]. The gene expression of ER_β_ is different from ER_α_ which plays an important role in the breast and uterine cancers. Due to the high similarity between the two isoforms, it is highly challenging to design or identify compounds which specifically target one subtype. Emerging data for ER indicates that the identification of selective agonist or antagonist for ER_β_ will help treat various cancers such as colon, breast, prostate, and lungs with lower side effects. Hence ER_β_ is considered as one of the emerging oncogene target.

Selective estrogen receptor modulator (SERM) is a drug or small molecule that acts as an agonist or antagonist by specifically binding to one of these two ER isoforms in the target tissues based on its specificity. The difference in binding activity to the two ER isoforms of a chemical is the metric for determination of chemicals that specifically bind for ER_α_ or ER_β_. The main mechanism of SERMs is to alter the estrogenic activity in the target tissues specificity. SERMs can selectively block the estrogens action in the breast cells and activate the estrogens action in bone, liver, and uterine cells. Hence, identification of selective estrogenic activity compound is an important task in drug discovery. Experimental identification of selective estrogenic activity compound is doable but very expensive and time consuming from a large pool of chemicals. Hence, *in silico* approaches for screening potential selective estrogenic activity compounds are in need. Some prediction models were developed using different *in silico* techniques such as pharmacophore model and molecular docking [10, 11].

Multiple quantitative structure activity relationship (QSAR) models have been developed for predicting ER_α_ binding activity using large sets of chemicals to ensure prediction reliability [12, 13]. Previously, we developed an ERα predictive model using the data set from Estrogenic Activity Database (EADB) and validated the model using a large data set from ToxCast [13]. Some QSAR models have been developed for predicting ER_β_ binding activity based on small sizes of chemicals of the particular scaffolds that cover a small chemical space [14–22]. Many factors affect the quality of a QSAR model, including the number of compounds and their chemical space coverage used for training, the algorithm used to train the model, and the method for validation of the model. More reliable QSAR models for ER_β_ binding activity need to be developed using large sets of diverse chemicals covering a large chemical space. Hence, in this study, we developed a QSAR model for predicting ER_β_ binding activity using large sets of chemicals which covers a wide range of chemical space.

DF (Decision Forest) algorithm [23–25] was used for development of the ER_β_ binding activity prediction model. The large set of chemicals and their ER_β_ binding activity data were collected from EADB [26, 27] and used to train the QSAR model. The compounds from the ToxCast data were used as an application data set to estimate concordance between the ER_β_ model predictions by traditional assay and the high-throughput screening results. The important molecular descriptors for ER_β_ binding were identified using the cross validations. Prediction confidence was analyzed to provide an additional metric for application of the ER_β_ QSAR model.

## RESULTS

### Cross validations

**Figure 1:**
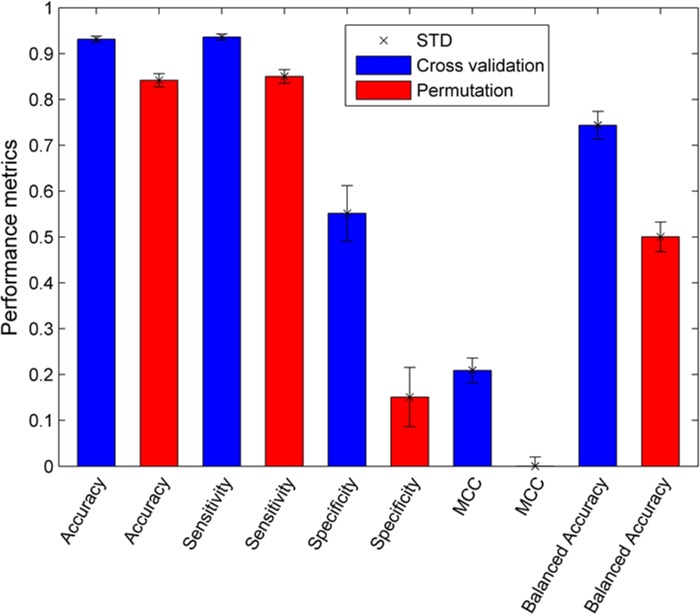
Performance of the 5-fold cross validations (blue bars) and permutation tests (red bars) Standard deviations were given on top of the bars. Performance metrics are indicated at the x-axis.

One thousand 5-fold cross validations were conducted to assess the goodness and robustness of the ER_β_ predictive models constructed from the training set. The results of the cross validations were summarized in Figure 1 (the blue bars). The average prediction accuracy, sensitivity, and specificity values for the 1000 5-fold cross validations were 93.1%, 93.6%, and 55.2%, respectively, indicating the ER_β_ predictive models performed well. Moreover, the standard deviations of prediction accuracy, sensitivity, and specificity in the 1000 cross validations were 0.6%, 0.7%, and 6.1%, respectively. The small fluctuation in the performance of the models in the cross validations demonstrated the robustness of the ER_β_ predictive models generated in the cross validations. Not surprisingly, the average specificity was low because much fewer ER_β_ non-binders than binders were included in the training data set. It is expected that specificity could be improved when more ER_β_ non-binders were identified for training the model.

### Permutation tests

The same performance metrics (prediction accuracy, sensitivity, and specificity) were calculated for the ER_β_ prediction models constructed in the 1000 permutation tests and were plotted as the red bars in Figure 1. The predictive accuracy, sensitivity, specificity, MCC, and balanced accuracy values for the 1000 permutation tests were 84.2%, 85.0%, 15.1%, 0.0%, and 50.0%, respectively, and their corresponding standard deviations were 1.5%, 1.5%, 6.5%, 2.0%, and 3.2%, respectively. The 0.0% MCC and 50.0% balanced accuracy obtained from the permutations matched the expectation for modeling random data sets, confirming the modeling process should be implemented correctly. The performance comparison between the 5-fold cross validations and permutation tests revealed that the 5-fold cross validations much outperformed the permutation tests, demonstrating the ER_β_ predictive models constructed from the training set had a good predictive power and were obtained unlikely solely by chance.

### Prediction confidence

**Figure 2:**
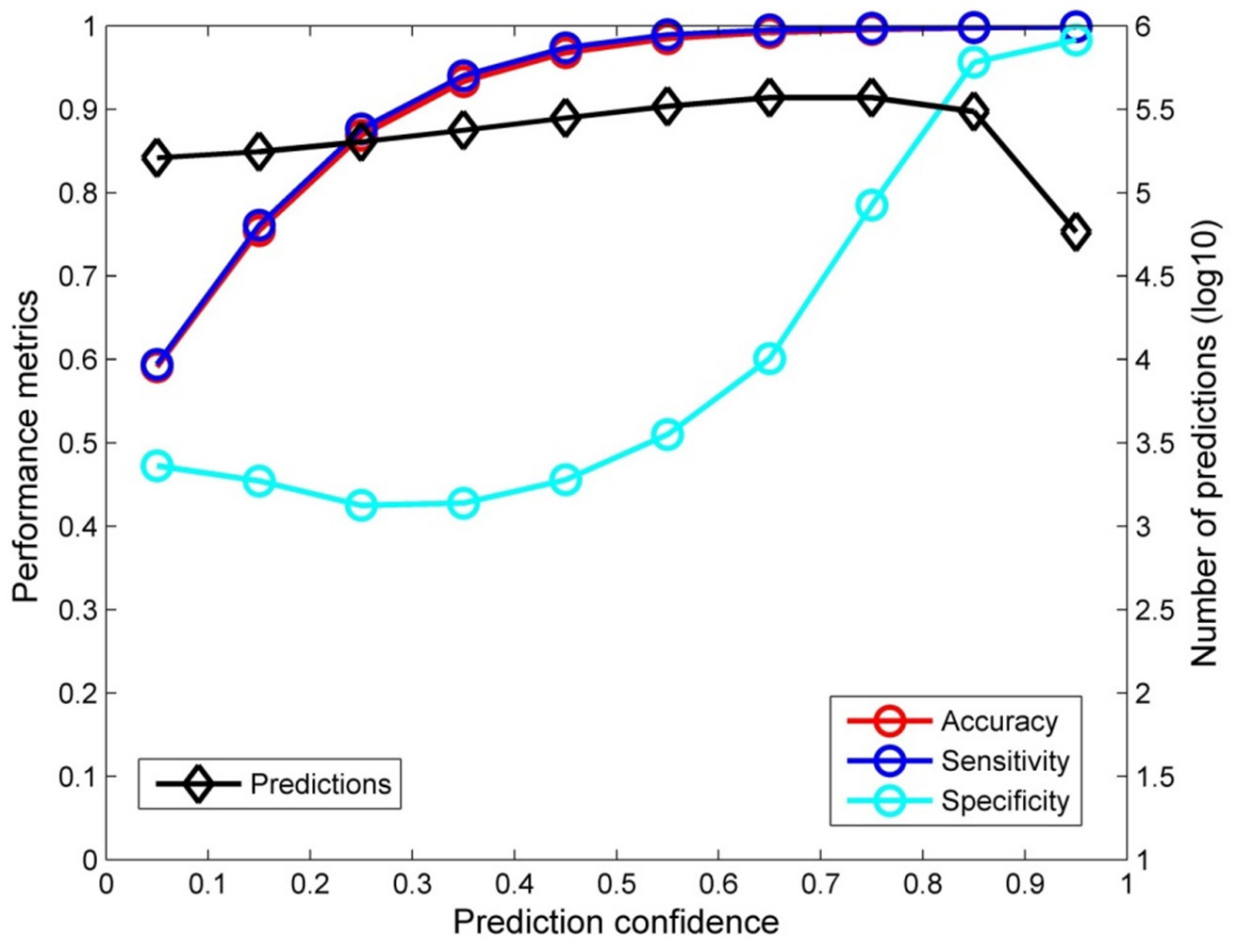
Confidence analysis result The accuracy (red circles), sensitivity (blue circles), and specificity (cyan circles) were given at the left y-axis and the numbers of predictions were plotted as black diamonds at the right y-axis for 10 even prediction confidence bins that were indicated at the x-axis.

The confidence analysis was performed on the predictions from the 5-fold cross validations. The predictions near evenly distributed in the 10 prediction confidence bins as shown by the black diamonds in Figure 2. When the prediction confidence increased, the performance of the corresponding predictions was also improved (accuracy, sensitivity, and specificity were all increased as depicted by the red, blue, and cyan circles, respectively). It is worth to note that when prediction confidence reached 0.8 or higher, the predictions were extremely accurate, close to 100% (95% for specificity). The prediction confidence analysis demonstrated that prediction confidence could be an additional metric for real application of the ER_β_ predictive model developed in this study.

### Informative descriptors

The 447 molecular descriptors in the training set were used in different predictive ER_β_ models generated in the 5-fold cross validations. The frequency (number of models) of each descriptor in the cross validations were calculated. These 447 molecular descriptors were then ranked by their frequency values. The top 18 descriptors were shown in Supplementary Table 1. Close up at the 18 descriptors revealed three physical chemical properties (atomic polarizability, electronegative, and van der Waals) were informative to the prediction models and should play major roles in ER_β_ binding of chemicals. The findings are consistent with our previously developed ER_α_ model that revealed molecular weight, van der Waals volume, polarizability, and aromatic rings are important for a chemical to bind ER_α_ [13]. Comparative analysis on structures of the two proteins showed that the binding sites are similar for ER_β_ and ER_α_. Off the 23 residues in the binding pockets, only two (ER_β_ M336 and I373 versus ER_α_ L384 and M421) are different (Supplementary Figure 1), confirming the similar chemical structural features for binding of the two receptors with a subtle difference (electronegative is important for ER_β_ binding and aromatic ring is vital to ER_α_ binding).

### Concordance between EADB and ToxCast

The experimental data of the 21 common compounds were compared to calculate the concordance between ToxCast ER_β_ dimerization assays activity and EADB ER_β_ binding activity. The 21 common compounds were all binders in EADB. ToxCast OT_ER_ER_β_ER_β__0480 ER_β_ dimerization assay showed 15 actives and 6 inactives (Table 1), while ToxCast OT_ER_ER_β_ER_β__1440 ER_β_ dimerization assay found 18 actives and 3 inactives (Table 2). If the experimental ER_β_ binding assay data in EADB were used to predict ToxCast ER_β_ dimerization data for the 21 common compounds, the prediction accuracy would be 71.4% and 85.7% for ToxCast OT_ER_ER_β_ER_β__0480 and OT_ER_ER_β_ER_β__1440 assays, respectively.

**Table 1:** Comparison between EADB and ToxCast data of common compounds

OT_ER_ER_β_ER_β__0480 Assay		Active	Inactive	Total
	**Binder**	15	6	21
**EADB**	**Non-binder**	0	0	0
	**Total**	15	6	21

**Table 2:** Comparison between EADB and ToxCast data of common compounds

OT_ER_ER_β_ER_β__1440 Assay		Active	Inactive	Total
	**Binder**	18	3	21
**EADB**	**Non-binder**	0	0	0
	**Total**	18	3	21

### Applications to ToxCast data

**Figure 3:**
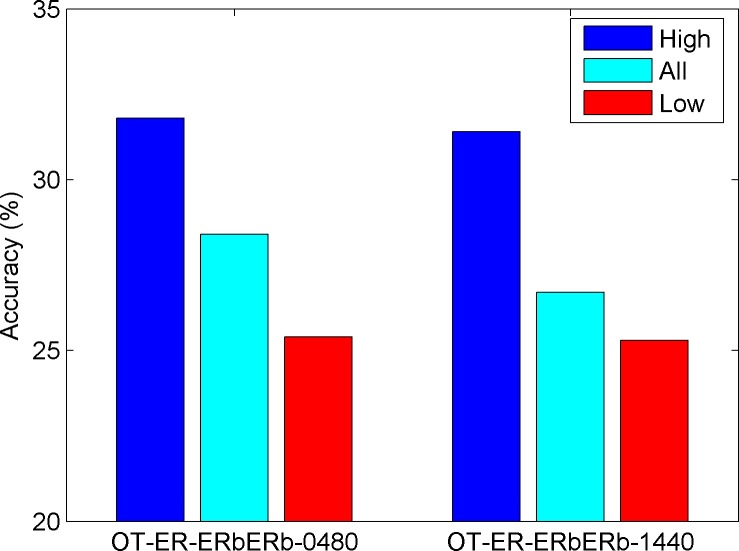
Result of application of the developed ER_β_ binding activity prediction model to the dimerization assays (labeled at the x-axis) in ToxCast Overall prediction accuracy (cyan) and prediction accuracy for high confidence predictions (blue) and low confidence predictions (red) were plotted as bars.

The 1805 compounds that were tested using ToxCast OT_ER_ER_β_ER_β__0480 assay and the 1800 compounds that were tested using ToxCast OT_ER_ER_β_ER_β__1440 assay were predicted using the ER_β_ binding prediction model generated based on EADB data. Overall prediction accuracy for ER_β_ dimerization activity using the model was low, 28.4% and 26.7% for OT_ER_ER_β_ER_β__0480 and OT_ER_ER_β_ER_β__1440, respectively. Prediction confidence analysis showed that high confidence predictions performed better than low confidence predictions (Figure 3). As the training data set had much less non-binders than binders, it is expected that the model would have better prediction on ER_β_ binders than non-binders (Figure 1). We examined the performance of the model on actives in ER_β_ dimerization. Of the 175 actives from OT_ER_ER_β_ER_β__0480 assay, 162 were predicted as ER_β_ binders (Table 3). The sensitivity 92.6% was very comparable to the sensitivity 93.6% in cross validations and 100% concordance for experimental actives and binders (Table 1). Of the 150 actives from OT_ER_ER_β_ER_β__1440 assay, 135 were predicted as ER_β_ binders (Table 4). Again, the sensitivity 90% was close to the cross validation and experimental data comparison (Table 2). The prediction on actives in ToxCast ER_β_ dimerization assays indicated that the ER_β_ predictive model trained using ER_β_ binding activity data from EADB could be extrapolated well to predict actives in ER_β_ dimerization. As expected, extrapolation of the model to inactives should be very cautious as very low specificity was yield in the applications: 21.5% and 21.0% for OT_ER_ER_β_ER_β__0480 and OT_ER_ER_β_ER_β__1440 assays, respectively.

**Table 3:** Predictions on OT_ER_ER_β_ER_β__0480 assay data

OT_ER_ER_β_ER_β__0480		Active	Inactive	Total
	**Binder**	162	1280	1442
**Prediction**	**Non-binder**	13	350	363
	**Total**	175	1630	1805

**Table 4:** Predictions on OT_ER_ER_β_ER_β__1440 assay data

OT_ER_ER_β_ER_β__1440		Active	Inactive	Total
	**Binder**	135	1304	1439
**Prediction**	**Non-binder**	15	346	361
	**Total**	150	1650	1800

## DISCUSSION

**Figure 4:**
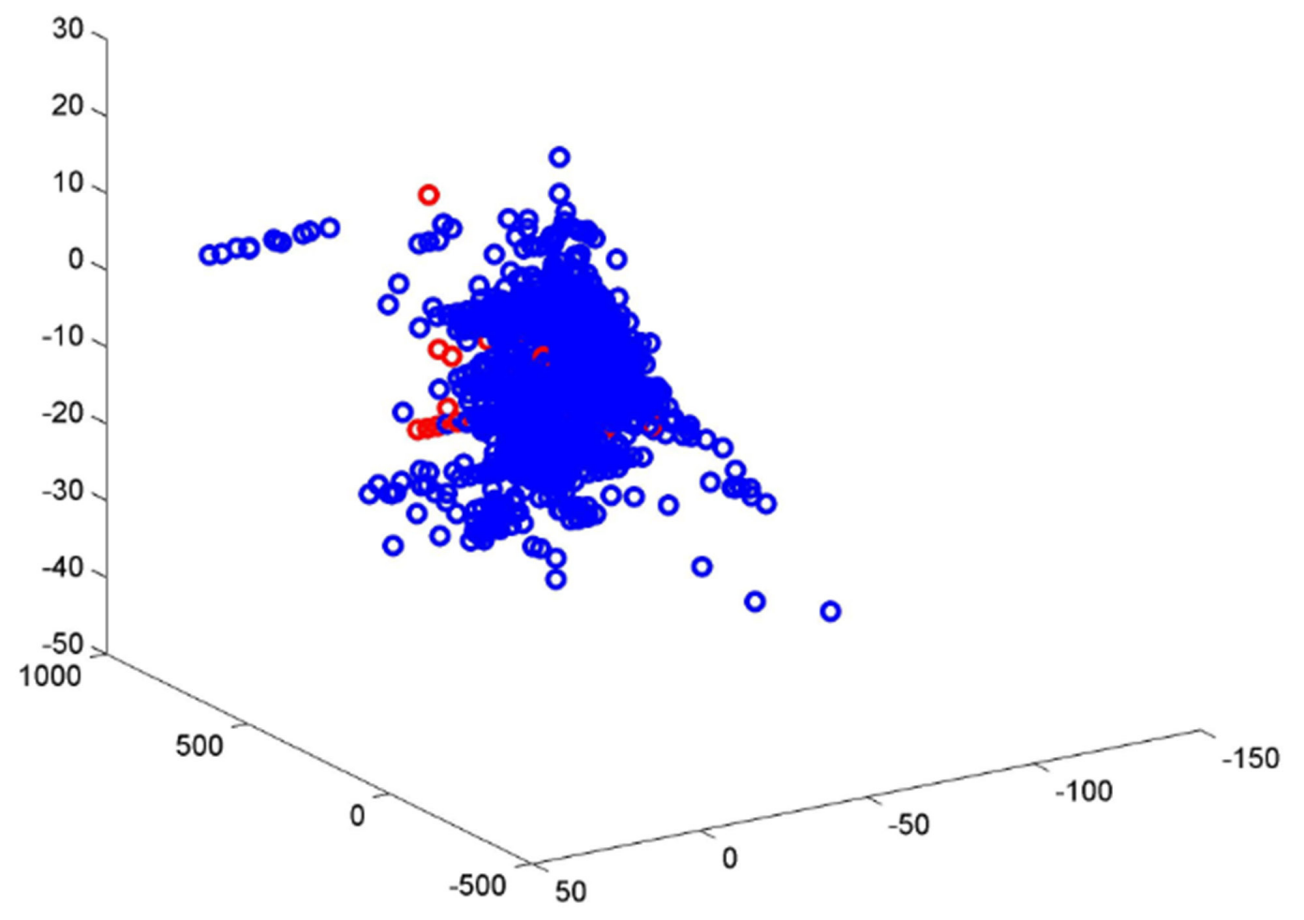
Chemical space of ER_β_ binders (blue circles) and non-binders (red circles)

The training set compounds were collected from the EADB with a well-defined end point (LogRBA) value which covers a wide range of ER_β_ binding activity. Multiple data points for the same compound were merged to a single value to classify the compound as an ER_β_ binder or non-binder. The training set has imbalanced distribution of ER_β_ binders (98.8%) and non-binders (1.2%). The high imbalance in samples is a challenging issue in machine learning. Even with the robust algorithm DF, the performance of the developed model is expected to be different between ER_β_ binders and non-binders. The analysis of performance of the 5-fold cross validations yielded a very high sensitivity (93.6%) and a low specificity (55.2%) are consistent with the expectation. The permutation tests results also confirmed that the bias to prediction on ER_β_ binders because of imbalanced samples even there were no signals in the data sets (randomly permuted samples). In addition to the difference in numbers of ER_β_ binders and non-binders, different chemical spaces covered by ER_β_ binders and non-binders were observed (Figure 4). The ER_β_ binders not only covered larger chemical space than non-binders but also had much higher density in the covered chemicals as shown in Figure 4. Much more chemical knowledge on ER_β_ binders than non-binders were learned by DF to generate the prediction model. It is not surprising that the prediction model had much better performance on ER_β_ binders than non-binders. Therefore, it is expected that including more non-binders in the training set would increase the chemical space of non-binders and improve performance of the prediction model based on a more balanced training set.

We not only demonstrated overall goodness of the ER_β_ binding prediction model but also conducted prediction confidence analysis to provide an additional metric that can be used in real applications of the model. The confidence analysis of the cross validation results (Figure 2) revealed that the ER_β_ predictive models showed high accuracy for the predictions with high confidence, while the predictions with low confidence did not perform well, especially for the predictions with confidence less than 0.3 (Figure 2). Therefore, in real applications of our ER_β_ binding activity prediction model, utilization of predictions with low confidence should be cautious.

We applied the ER_β_ binding activity prediction model to the ER_β_ related active data in ToxCast to assess the capability of extrapolation of the model to other ER_β_ related endpoints such as the two ER_β_ dimerization assays. The application results revealed that the ER_β_ predictive model trained using the binding activity data from EABD was able to predict most of the actives from the two ER_β_ dimerization assays (Tables 3 and 4). However, as expected the ER_β_ predictive model did not show a good performance for predicting inactives from two ER_β_ dimerization assays. One of the reasons for the poor performance of the ER_β_ predictive model on the inactive chemicals in the dimerization assays might be due to the difference in the assays for the training data and application data. The gene expression modulated by ER_β_ is a cascade of events: ligand binding ER_β_ induces ER_β_ dimerization, translocation of ER_β_ER_β_ dimers to the nucleus, and recognition of Estrogen Response Elements (EREs) on DNA. Binding assays and dimerization assays measure a chemical activity on two different events in the function mechanism. Therefore, extrapolation of the developed model to prediction of inactives from other ER_β_ related endpoints such as dimerization should be very cautious. The model could be improved by adding more inactive compounds in training to increase chemical space for inactivs.

Comparison between the cross validations and the applications revealed that extrapolation to ER_β_ dimerization assay data yield less accurate predictions than the cross validations, especially for the predictions on the inactives. The reasons for that could be (i) all the ER_β_ binders play a role in the dimerization of ER_β_,(ii) the EADB reported binders are not necessary to lead a dimerization signal to be observed in the ER_β_ dimerization assays, and (iii) the traditional ER_β_ binding assays are different from the high-throughput screening assays.

The ER_β_ binding activity prediction model was developed and its prediction performance and extrapolation to other ER_β_ related endpoints data were demonstrated. Comparison of ER_β_ binding activity predicted by the model developed in this study with ER_α_ binding activity estimated using our previous ER_α_ model [13] could identify chemicals that specifically bind ER_α_ or ER_β_, facilitating drug discovery and development.

## MATERIALS AND METHODS

### Study design

**Figure 5:**
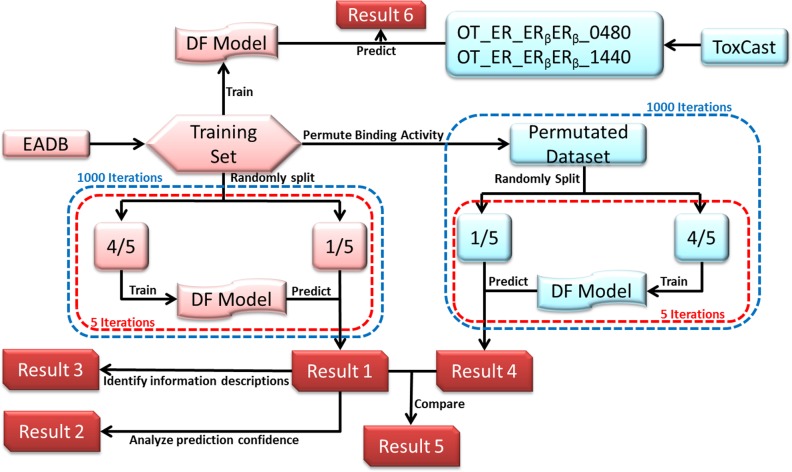
Study design Data from EADB was used as a training set. Mold^2^ in-house software was used to generate the molecular descriptors for all compounds. Decision Forest (DF) classification method was used to generate ER_β_ predictive models. Five-fold cross validations (red dashed boxes) were conducted 1000 times (blue dashed boxes) in cross validations (left panel and Result 1) and permutation tests (right panel and Result 4). Based on the 5-fold cross validations, prediction confidence analysis was conducted (Result 2) and informative descriptor were identified (Result 3). ToxCast data of 2 dimerization assays were used assess the capability of the model (Result 6).

The study design was depicted in Figure 5. The data sets were collected from two large data sources: EADB [26] and ToxCast databases [28, 29]. The compounds from EADB were used to develop the ER_β_ predictive model using DF. The ER_β_ binding affinity data from EADB (recorded as logarithmic value of relative binding activity (logRBA)) were used to determine ER_β_ binders or non-binders for the training compounds. The activity values for 1812 compounds from two ER_β_ related assays in the ToxCast were used to assess concordance between EADB and ToxCast data as well as to estimate the performance of the QSAR model for predicting ToxCast results. The common compounds contained in both EADB and ToxCast databases were used for assessing the concordance. The compounds only assayed in ToxCast were used for estimating the goodness of extrapolation of the QSAR model to high-through screening assays. The Mold^2^ software [30] was used to generate the molecular descriptors for compounds in the training and application sets. The generated molecular descriptors were preprocessed to remove the less informative descriptors. The preprocessed training data set were used to develop the ER_β_ predictive model using DF [2, 23, 31–33]. Robustness of performance of the ER_β_ predictive models were estimated through 5-fold cross-validations. Prediction confidence of the 5-fold cross-validation results was analyzed. The critical molecular descriptors to ER_β_ binding were identified by the analysis of their frequency in the models during the cross validations. Predictive power and chance correlation of the models were assessed using permutation tests. The developed model was then used to predict ER_β_ activity of chemical assayed in ToxCast.

### Data sets preparations

#### Training data set

**Figure 6:**
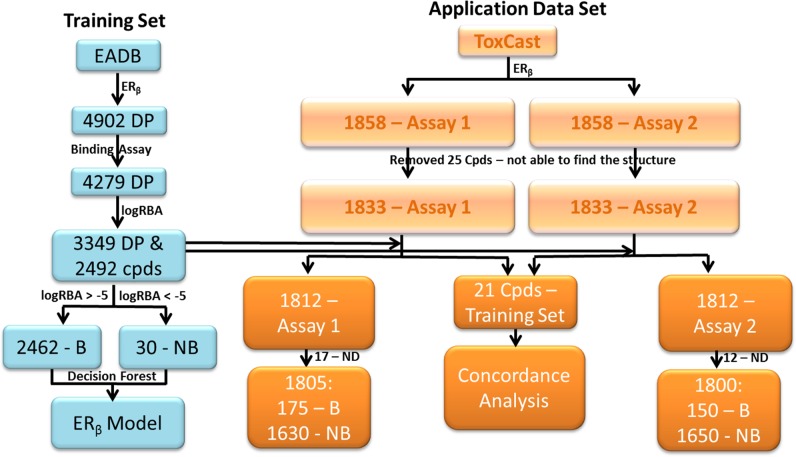
Data sets used in this study Training data set was collected from EADB (left blue panel). Common data set contained 21 chemicals that had data in both ToxCast and EADB (middle of the right light brown panel). Application data sets were extracted from the two ER_β_ dimerization assays data in ToxCast (the right light brown panel) and not contained in the training data set. Assay 1- OT_ER_ER_β_ER_β__0480 and Assay 2 – OT_ER_ER_β_ER_β__1440. B – Binder and NB – Non-binder.

The training set compounds were extracted from the FDA's EADB [24, 26, 27, 31, 33–36]. The ER_β_ binding activity (binder or non-binder) for 2492 compounds were determined based on their logRBA (Relative Binding Affinity) values. The binding assays measure ER_β_ binding affinity of chemicals. Most of the ER_β_ binding activity data in EADB are determined using competitive binding assays. A competitive binding assay works by measuring how well a chemical competes with the radiolabeled ligand for the receptor ER_β_ and the binding affinity of the chemical is given as an IC_50_ value. For the experiments with the same reference chemical tested, the binding affinity IC_50_ values were converted into logRBA values. A compound was assigned as an ER_β_ binder if its logRBA value is equal to or greater than -5; otherwise the compound was determined as a non-binder (Figure 6). If a compound contains more than one logRBA value, it was assigned as an ER_β_ binder or non-binder based on the consensus. Of the 2492 training compounds, 2462 were determined as ER_β_ binders and only 30 were non-binders. The determination of ER_β_ binder or non-binder for the 2492 compounds was given in Supplementary Table 2.

#### Application data sets

There are 1858 compounds in the ToxCast database that were tested by ER_β_ dimerization assays (http://epa.gov/ncct/toxcast/data.html, accessed on March 2, 2016) [28, 29, 37, 38] such as OT_ER_ER_β_ER_β__0480 and OT_ER_ER_β_ER_β__1440. These two assays use a yellow fluorescent protein reporter that is dissected into two fragments and fused to two ER_β_ to interact within a signaling complex. The reporter is fully assembled when ER_β_ dimerize. Therefore, when the ER_β_ molecules are physically separated, no signal is detected; however, when two ERβ are in close physical proximity, the two fragments from the reporter interact to elicit a signal. Changes in signal intensity and location are used to measure dimerization activity of a chemical. Twenty-five compounds which do not have CAS numbers were removed. Twenty one compounds were contained in both the training set and ToxCast. They were used for assessing the concordance between the ER_β_ binding assays in EADB and the high-throughput screening ER_β_ dimerization assays in ToxCast. After removed the 21 compounds, the remaining compounds and their ER_β_ dimerization assay data [39] from ToxCast were used as the application data sets for estimating the extrapolation capability of the ER_β_ binding activity prediction model. Among the 1812 compounds that were not included in the training set, 17 and 12 compounds were reported as not determined in OT_ER_ER_β_ER_β__0480 and OT_ER_ER_β_ER_β__1440 assays, respectively. The compounds in the application data sets were classified as active if their AC_50_ values are recorded in the ToxCast database; otherwise they were determined as inactive. Finally, 1805 (175 binders and 1630 non-binders) compounds for OT_ER_ER_β_ER_β__0480 and 1800 (150 binders and 1650 non-binders) compounds for OT_ER_ER_β_ER_β__1440 were used in the application data sets.

### Mold^2^: Descriptor generation

Mold^2^ (http://www.fda.gov/ScienceResearch/BioinformaticsTools/Mold2) software [30] was used to generate molecular descriptors for the compounds in the training and application sets. Compounds in the SDF files were input into Mold^2^. Totally, 777 molecular descriptors were generated for each compound. Mold^2^ generates one-dimensional (e.g., molecular weight) and two dimensional (e.g., structural and bond information) descriptors. The generated molecular descriptors were pre-processed to remove the less informative descriptors (i.e., the same value for most of the chemicals in the training set). Out of 777 molecular descriptors, 330 descriptors are removed as they were considered as less informative (Supplementary Figure 2). Finally, 447 descriptors were subjected to the DF algorithm to generate the ER_β_ predictive model.

### DF

DF is a powerful algorithm that combines multiple decision tree models to generate a consensus predictive model with high accuracy [23–25]. Each decision tree model is generated using a different set of molecular descriptors. The algorithm of DF consists of four steps. Initially, it constructs an individual decision tree using a pool of molecular descriptors; the molecular descriptors used in the previously generated decision tree model are then removed from the pool of molecular descriptors; the above two steps are repeated until no improvement can be achieved by adding more decision tree models; and finally, it combines all the decision tree models to make a final predictive model. DF is different from the Random Forests algorithm [40]. DF uses less decision trees that are constructed using all samples and variables. Therefore the trees are deep and accurate. In contract, Random Forests combines a large number of decision trees that are built using part of samples through bootstrapping and a small fraction of variables randomly selected. Thus, the trees are shallow and not accurate. The diversity of decision trees that is achieved by using different variables is the key to DF, while Random Forests ensures contributions of independent variables by randomization that requires a large number of such shallow trees.

### Performance metrics

The performance of the ER_β_ predictive model can be measured using different metrics. Prediction accuracy, sensitivity, selectivity, Matthews's correlation coefficient (MCC), and balanced accuracy were used in this study. The above mentioned metrics are computed using the following equations:

$$\text{Accuracy}=\frac{\text{TP}+\text{TN}}{\text{TP}+\text{TN}+\text{FP}+\text{FN}}$$

$$\text{Sensitivity}=\frac{\text{TP}}{\text{TP}+\text{FN}}$$

$$\text{Specificity}=\frac{\text{TN}}{\text{TP}+\text{FN}}$$

$$\text{MCC}=\frac{\text{TP*TN}-\text{FP*FN}}{\sqrt{\left(\text{TP}+\text{FP}\right)\left(\text{TP}+\text{FN}\right)\left(\text{TN}+\text{FP}\right)(\text{TN}+\text{FN})}}$$

Balanced accuracy = (Sensitivity + Specificity) / 2

In the above equations, TP indicates true positives (number of actual binders are predicted as binders); TN represents true negatives (number of actual non-binders are predicted as non-binders); FP means false positives (number of actual non-binders are predicted as binders); and FN is false negatives (number of actual binders are predicted as non-binders).

### Cross validation

Five-fold (5-fold) cross validations were used to evaluate the performance of ER_β_ predictive models. In a 5-fold cross validation, the training set was randomly divided into five equal subsets. Four subsets were used to generate a DF model and the remaining one subset was used to test the generated DF model. This process was repeated for each of the five subsets once and only once. The prediction results from the five subsets were then average to assess the prediction performance of the five DF models. To avoid possible chance correlation in the random division of a data set into five subsets, the 5-fold validation was repeated for 1000 times using different random divisions to reach a statistically reliable validation.

### Permutation test

Permutation tests were used to estimate the predictive power of the generated models. In a permutation test, the ER_β_ binding activity data in the training data set were randomly scrambled to generate a permuted data set. A 5-fold cross validation test was performed on the permuted data set. The accuracy, specificity, and sensitivity of the 5-fold cross validation test were calculated to measure the chance correlation. To reach a statistically reliable estimation, this process was repeated for 1000 times to generate the 1000 permuted data sets for running 5-fold cross validations.

### Prediction confidence analysis

Prediction confidence analysis was carried out not only to classify the compounds as ER_β_ binder and non-binder but also to estimate the probability of the compounds to be an ER_β_ binder or non-binder. Hence, the models were assessed based on not only the overall prediction performance but also the relationship between the prediction confidence levels and their performance. The prediction confidence value was calculated using the below equation.

$$\text{Confidence }=\frac{\left|\text{P}-0.5\;\right|}{0.5}$$

Where P represents the probability of a chemical being an ER_β_ binder (0.5 <= P <=1) or non-binder (0 < P < 0.5). Prediction confidence is a value between 0 and 1. The larger the prediction confidence is, the more confident the prediction is. To conduct confidence analysis, the prediction confidence was calculated for each of the predictions from the 1000 iterations of 5-fold cross validations at first. The predictions were then put into 10 even bins based on their prediction confidence values. Prediction performance metrics were finally calculated for the predictions in each of the prediction confidence bins according to their actual and predicted ER_β_ binding activity.

### Identification of informative descriptors

The importance of molecular descriptors in ER_β_ binding was estimated based on the frequency distribution of the descriptors used in the models in the 5-fold cross validations. A frequency value in the models from 5-fold cross validations was calculated for each of the molecular descriptors. The molecular descriptors were then ranked based on their frequency values. The descriptors that were used in more than 90% of the models were finally selected as the informative descriptors for the ER_β_ models.

### Concordance between EADB and ToxCast

The two ER_β_ dimerization assays from ToxCast reported the estrogenic activity for >1800 compounds. Among the >1800 compounds, 21 had ER_β_ binding data in EADB. The ER_β_ binding data in EADB and the estrogenic activity data from high-throughput screening assays in ToxCast (Supplementary Table 3) were compared for the 21 common compounds to assess the correlation between the two types of assays (traditional and high-throughput screening assays). This analysis could help to understand how well the ER_β_ binding predictive model would be applied to data from different assays. The balanced accuracy was calculate for the two different ER_β_ dimerization assays in ToxCast relative to EADB, where EADB was used as a primary metrics to measure the activity data agreement between EADB and ToxCast assays.

### Applications

The application data sets were used to assess the goodness of the ER_β_ binding activity prediction model in extrapolation to predicting chemicals for their high-throughput screening ER_β_ dimerization assays activity. The application data sets contain 1805 and 1800 compounds for OT_ER_ER_β_ER_β__0480 and OT_ER_ER_β_ER_β__1440 ER_β_ dimerization assays from ToxCast. The constructed ER_β_ binding activity predictive model was used to predict ER_β_ binders and non-binders for the chemicals in the application data sets. The accuracy, sensitive, selectivity, and balanced accuracy were calculated to assess the goodness of the ER_β_ binding activity predictive model, when the model was extrapolated to predicting outcome from high-throughput screening ER_β_ dimerization assays.

### Disclaimer

The views expressed in this manuscript do not necessarily represent those of the U.S. Food and Drug Administration.

## CONCLUSIONS

The ER_β_ predictive model was developed and cross validated using a large data set from EADB. The cross validations demonstrated the predictive power of the ER_β_ predictive model. The prediction confidence provides an additional metric for application of the ER_β_ predictive model. The important molecular descriptors linked to the knowledge of ligand binding to ER were identified. Applications of the developed model to ER_β_ dimerization assays data in ToxCast demonstrated the capability of extrapolation to other ER_β_ related endpoints data. Combination of the model developed in this study with our previous ER_α_ binding activity prediction model could help to design or identify the selective compounds for ER_β_ and ER_α_ which would be crucial in drug discovery and safety evaluation.

## SUPPLEMENTARY MATERIALS TABLES AND FIGURES



## Abbreviations

ER: Estrogen receptor
SERMs: Selective estrogen receptor modulators
DF: Decision forest
EADB: Estrogenic Activity Database
NTD: N-terminal domain
DBD: DNA-binding domain
LBD: Ligand-binding domain
CTD: C-terminal domain
AF1: Activation function 1
AF2: Activation function 2
ERE: Estrogen response element
SERM: Selective estrogen receptor modulator
QSAR: Quantitative structure activity relationship
MCC: Matthew's correlation coefficient
TP: True positive
TN: True negative
FP: False positive
FN: False negative
