# Supplementary material for: Development of estrogen receptor beta binding prediction model using large sets of chemicals
Source: Oncotarget. 2017 Oct 10;8(54):92989–3000. doi: 10.18632/oncotarget.21723 (PMC5696238; doi:10.18632/oncotarget.21723)
Supplement: Supplementary file 1 [file oncotarget-08-92989-s001.pdf]

Development of estrogen receptor beta binding prediction model using large sets of chemicals

SUPPLEMENTARY MATERIALS

Supplementary Table 1: Top 18 informative molecular descriptors

Mold^2 ID	Description	Frequency
D508	Moran autocorrelation - lag 6 / weighted by atomic polarizabilities	32556
D468	Geary autocorrelation - lag 6 / weighted by atomic Sanderson electronegativities	22231
D507	Moran autocorrelation - lag 5 / weighted by atomic polarizabilities	19917
D506	Moran autocorrelation - lag 4 / weighted by atomic polarizabilities	16593
D562	Lowest eigenvalue n. 7 of Burden matrix / weighted by atomic polarizabilities	14943
D487	Moran autocorrelation - lag 1 / weighted by atomic van der Waals volumes	14890
D535	Lowest eigenvalue n. 4 of Burden matrix / weighted by atomic masses	14232
D353	Molecular multiple path count of order 05	14183
D263	Graph vertex complexity index	14090
D518	Topological charge index of order 8	13673
D523	Mean topological charge index of order3	13236
D250	Path/walk 5 - randic shape index	12450
D567	Highest eigenvalue n. 4 of Burden matrix / weighted by atomic masses	11998
D464	Geary autocorrelation - lag 2 / weighted by atomic Sanderson electronegativities	10701
D254	Radial centric information index	10504
D462	Geary autocorrelation - lag 8 / weighted by atomic van der Waals volumes	10350
D497	Moran autocorrelation - lag 3 / weighted by atomic Sanderson electronegativities	10224
D534	Lowest eigenvalue n. 3 of Burden matrix / weighted by atomic masses	9763

Supplementary Table 2: Training data set

See Supplementary File 1

Supplementary Table 3: Compounds contained in both EADB and ToxCast

EADB_ID	ToxCast_ID	CAS	Name	EADB*	OT_ER_ ER$_{\beta}$ER$_{\beta}$_0480&	OT_ER_ ER$_{\beta}$ER$_{\beta}$_1440&
5	C68392358	68392-35-8	4-hydroxytamoxifen	1	1	1
11	C104405	104-40-5	4-nonylphenol	1	1	1
41	C1806264	1806-26-4	4-octylphenol	1	1	1
66	C98544	98-54-4	4-tert-butylphenol	1	1	1
17	C80057	80-05-7	Bisphenol A	1	1	1
67	C480400	480-40-0	Chrysin	1	0	1
455	C50226	50-22-6	Corticosterone	1	0	0
10	C486668	486-66-8	Daidzein	1	1	1
6	C56531	56-53-1	Diethylstilbestrol	1	1	1
135	C474862	474-86-2	Equilin	1	1	1
13	C50271	50-27-1	Estriol	1	1	1
7	C53167	53-16-7	Estrone	1	1	1
53	C525826	525-82-6	Flavone	1	0	0
3	C446720	446-72-0	Genistein	1	1	1
26	C143500	143-50-0	Kepone	1	0	0
209	C72333	72-33-3	Mestranol	1	1	1
285	C68224	68-22-4	Norethindrone	1	1	1
110	C72559	72-55-9	P,P′-DDE	1	1	1
16	C57830	57-83-0	Progesterone	1	0	1
35	C117395	117-39-5	Quercetin	1	0	1
2	C10540291	10540-29-1	Tamoxifen	1	1	1

*1 indicates binder; & 1 represents active and 0 means inactive.

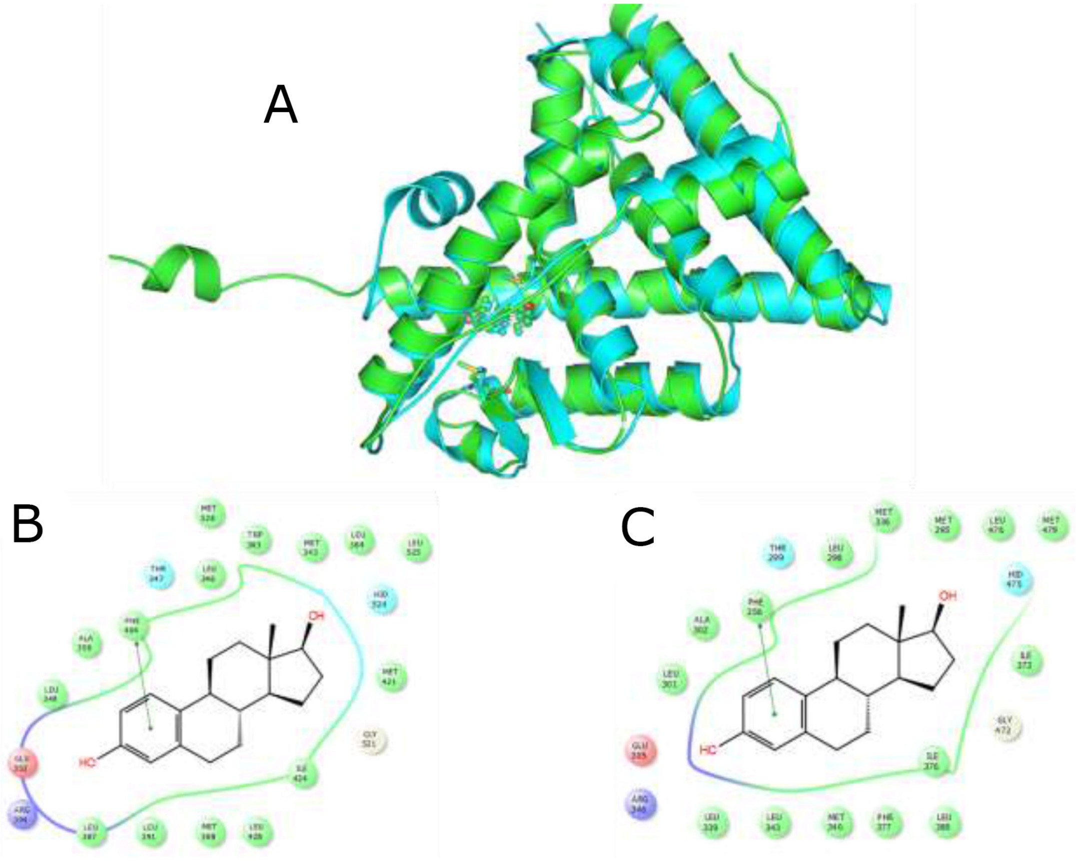

Supplementary Figure 1: (A) Overlay of the cartoon representation of the ER$_{\beta}$ (Cyan) and ERα (Green). The estrogen ligand was shown in ball and stick. The two different residues in the ligand binding site of ER$_{\beta}$ and ER$_{\alpha}$ were shown in stick representation. **(B)** Interaction between the ER$_{\alpha}$ and estrogen. **(C)** Interaction between the ER$_{\beta}$ and estrogen.

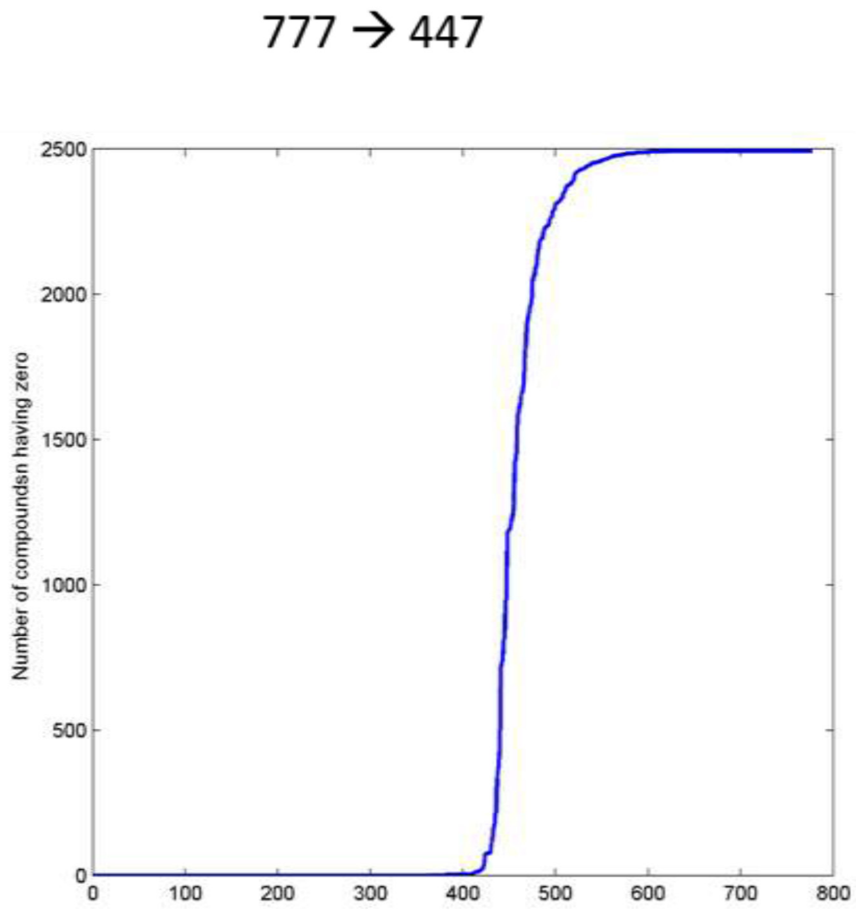

Supplementary Figure 2: 777 molecular descriptors generated using Mold². The less informative descriptors which had the same value for most of the compounds in the training set were removed. The x-axis gives the rank of descriptors that have zero values which is indicated at the y-axis.
